# Aquaporins as Targets of Dietary Bioactive Phytocompounds

**DOI:** 10.3389/fmolb.2018.00030

**Published:** 2018-04-18

**Authors:** Angela Tesse, Elena Grossini, Grazia Tamma, Catherine Brenner, Piero Portincasa, Raul A. Marinelli, Giuseppe Calamita

**Affiliations:** ^1^Centre National de La Recherche Scientifique, Institut National de la Santé et de la Recherche Médicale, l'Institut du Thorax, Universitè de Nantes, Nantes, France; ^2^Laboratory of Physiology, Department of Translational Medicine, University East Piedmont, Novara, Italy; ^3^Department of Biosciences, Biotecnhologies and Biopharmaceutics, University of Bari “Aldo Moro”, Bari, Italy; ^4^Institut National de la Santé et de la Recherche Médicale UMR-S 1180-LabEx LERMIT, Université Paris-Sud, Université Paris-Saclay, Châtenay Malabry, France; ^5^Clinica Medica “A. Murri”, Department of Biomedical Sciences and Human Oncology, Medical School, University of Bari “Aldo Moro”, Bari, Italy; ^6^Instituto de Fisiología Experimental, CONICET, Facultad de Ciencias Bioquímicas y Farmacéuticas, Universidad Nacional de Rosario, Rosario, Argentina

**Keywords:** aquaporin membrane channels, functional foods, nutraceutics, epigenetics, gut microbiota, antioxidants, anti-inflammatory, chronic diseases

## Abstract

Plant-derived bioactive compounds have protective role for plants but may also modulate several physiological processes of plant consumers. In the last years, a wide spectrum of phytochemicals have been found to be beneficial to health interacting with molecular signaling pathways underlying critical functions such as cell growth and differentiation, apoptosis, autophagy, inflammation, redox balance, cell volume regulation, metabolic homeostasis, and energy balance. Hence, a large number of biologically active phytocompounds of foods have been isolated, characterized, and eventually modified representing a natural source of novel molecules to prevent, delay or cure several human diseases. Aquaporins (AQPs), a family of membrane channel proteins involved in many body functions, are emerging among the targets of bioactive phytochemicals in imparting their beneficial actions. Here, we provide a comprehensive review of this fast growing topic focusing especially on what it is known on the modulatory effects played by several edible plant and herbal compounds on AQPs, both in health and disease. Phytochemical modulation of AQP expression may provide new medical treatment options to improve the prognosis of several diseases.

## Introduction

Growing evidence from epidemiological, *in vivo, in vitro*, and clinical trial results indicate that the plant-based food can reduce or prevent the risk of chronic diseases such as cardiovascular disease, arterial hypertension, diabetes mellitus, and cancer due to presence of biologically active plant compounds or phytochemicals. Several classes of phytochemicals from edible plants and herbs exist (Steinmetz and Potter, 1991) and exert beneficial effects in disease prevention and in reducing the incidence of certain chronic diseases. The mechanisms modulate the cell signaling pathways underlying inflammation, oxidative stress, metabolic disorder, apoptosis, and so forth (Maraldi et al., 2014).

This review provides an update on the involvement of Aquaporins (AQPs), a family of membrane channel proteins with important role in many body functions, in the beneficial effects imparted by food polyphenols and herbal phytocompounds, both in health and disease.

## Aquaporins, a family of membrane channels widely distributed in human tissues

**Figure 1:**
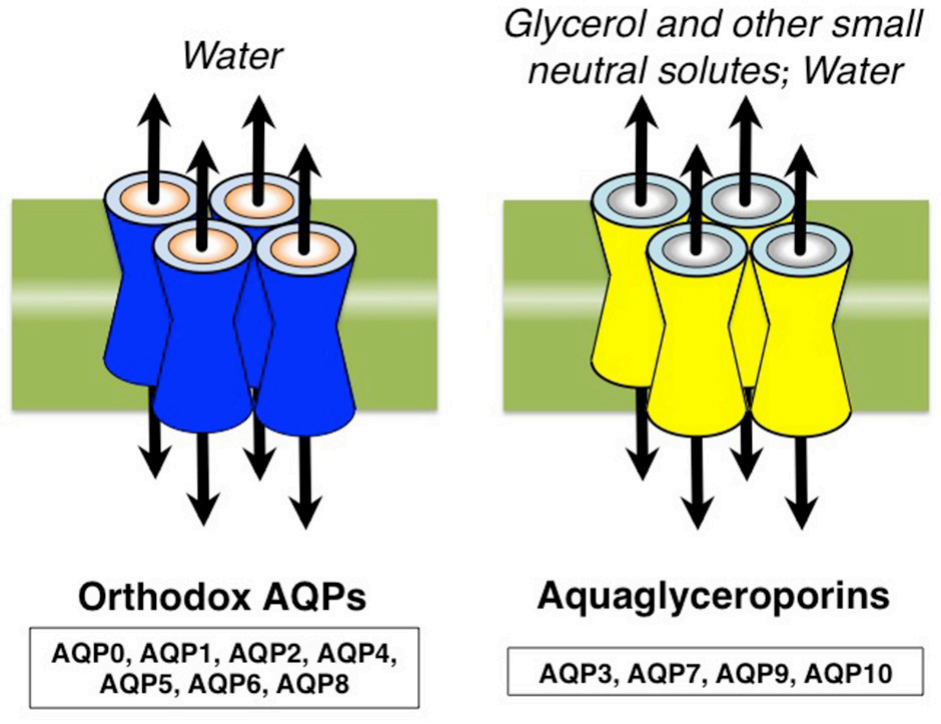
Mammalian aquaporins are grossly subdivided in *orthodox aquaporins* (AQP0, AQP1, AQP2, AQP4, AQP5, AQP6, and AQP8) and *aquaglyceroporins* (AQP3, AQP7, AQP9, and AQP10) depending on their ability to conduct only water or glycerol and some other small neutral solutes, in addition to water, respectively. Two of the 13 AQPs found in mammals, AQP11 and AQP12, are called *unorthodox aquaporins* as they show marked distinctions in terms of evolutionary pathway. Some AQPs also express conductance to gases of physiological relevance.

**Figure 2:**
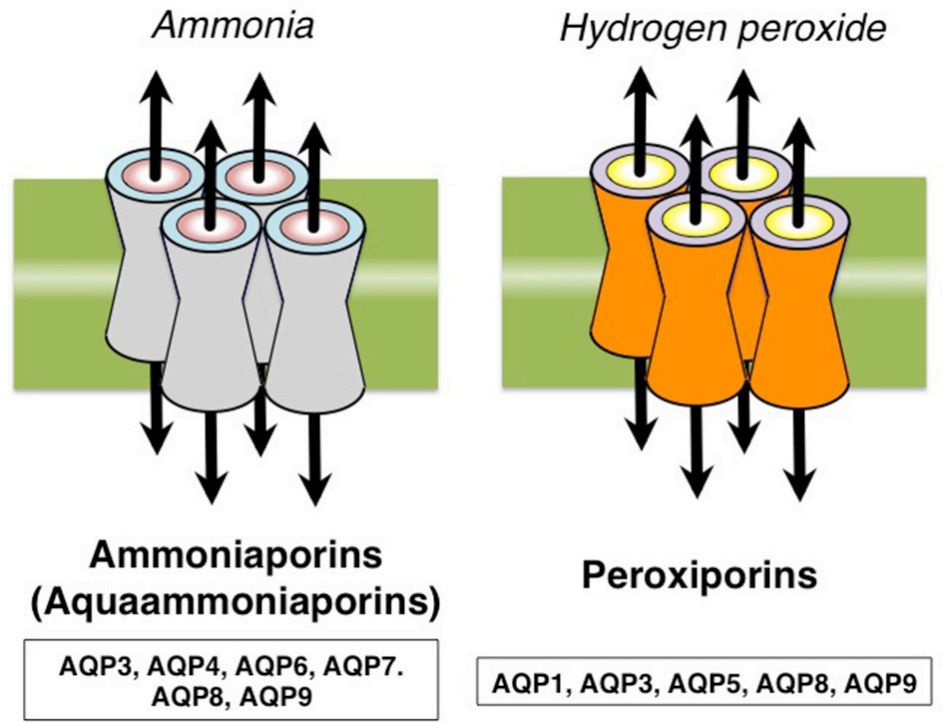
Some AQPs also allow transport of ammonia (AQP3, AQP4, AQP6, AQP7, AQP8, and AQP9), particularly AQP8, and/or hydrogen peroxide (AQP1, AQP3, AQP5, AQP8, and AQP9) and are called *ammoniaporins* (or *aquaammoniaporins*) and *peroxiporins*, respectively.

Aquaporins (AQPs) are channel proteins largely expressed in living organisms mediating the transport of water and some anaelectrolytes across biological membranes (Agre, 2004). The 13 AQPs (AQP0-12) expressed in mammals are summarily grouped into *orthodox aquaporins* (AQP0, AQP1, AQP2, AQP4, AQP5, AQP6, and AQP8) and *aquaglyceroporins* (AQP3, AQP7, AQP9, AQP10), depending on their ability to conduct only water or water and neutral solutes, particularly glycerol, respectively (Figure 1). AQP11 and AQP12 are often grouped as *unorthodox aquaporins* due to their distinct evolutionary pathway and transport properties (Ishibashi et al., 2009). Some AQPs are also able to conduct ammonia (AQP3, AQP4, AQP6, AQP8, and AQP9) and/or hydrogen peroxide (AQP1, AQP3, AQP5, AQP8, and AQP9) and, for these biophysical properties, are also denoted as *ammoniaporins* (or *aquaammoniaporins*) (Jahn et al., 2004) and/or *peroxiporins* (Geyer et al., 2013; Almasalmeh et al., 2014; Rodrigues et al., 2016; Watanabe et al., 2016) (Figure 2). Moreover, some AQPs also allow permeation of gases of physiological importance such as CO_2_, NO or O_2_ (Nakhoul et al., 1998; Herrera et al., 2006; Wang et al., 2007). Expression, transport properties (Agre, 2004), and pharmacological gating (Soveral and Casini, 2017) of AQPs are object of strong interest and intense investigation in all body districts and a number of important roles have been already described, both in health and clinical disorders.

## Dietary polyphenols and aquaporins

The class of polyphenols is characterized by the presence of phenol units in their chemical structure. Polyphenols are the largest group of phytochemicals, and many of them exist in edible plants (Maraldi et al., 2014). Foods enriched in polyphenols were found to exert a wide spectrum of protective effects (i.e., hypolipidemic, anti-oxidative, anti-proliferative, anti-apoptotic, and anti-inflammatory) with the benefit of reducing the prognosis and onset of disease progression (for review see Upadhyay and Dixit, 2015). So far, more than 8,000 phenolic structures have been identified in vegetables, fruits, olive oil, and wine. Due to their diversity and food distribution the latest classification subdivides polyphenols in phenolic acids, curcuminoids, flavonoids, chalcones, stilbenes, lignans, and isoflavonoids (González-Castejón and Rodriguez-Casado, 2011; Upadhyay and Dixit, 2015). Bioactive polyphenols also influence the expression and biophysical properties of mammalian AQPs (Zhang et al., 2014; Fiorentini et al., 2015; Cataldo et al., 2017). The AQPs modulated by polyphenols and related health benefits are summarized in Table 1.

**Table 1:** Polyphenolic modulation of AQPs and related beneficial effects.

**Polyphenol**	**Functional derivative**	**Modulated AQP**	**Beneficial effect**	**References**
Curcuminoids	Curcumin	Choroid plexus AQP1 ↓ (brain lateral ventricle)	Reduction of intracranial pressure in brain injury (rat model)	Nabiuni et al., 2013
		CaOV3 AQP3 ↓ (ovarian cancer cell line)	Inhibition of ovarian cancer cell migration (*in vitro*)	Ji et al., 2008; Terlikowska et al., 2014
		Brain AQP4 and AQP9 ↓	Reduction of brain edema (rodent model)	Yu et al., 2012; Zhang et al., 2014; Zu et al., 2014; Wang et al., 2015
		HeLa cells AQP (ND)	Hydrogen peroxide elimination (*in vitro*)	Pellavio et al., 2017
Flavonoids	Pinocembrin	Brain AQP4 ↓	Reduction of cerebral edema due to ischemia (rat model)	Gao et al., 2010
	Chrysin	Skin AQP3 ↑	Protection against UV-induced skin damages (*in vitro*)	Wu et al., 2011
	Quercetin	Microglial AQP4 ↓	Amelioration of diabetic retinal edema (rat model)	Kumar et al., 2014
		Salivary gland, lung AQP5 ↑	Amelioration of impaired salivation after irradiation and IAV-induced lung injury (mouse models)	Takahashi et al., 2015; Yu et al., 2016a
		HeLa cells AQP1, AQP3, AQP8, AQP11	Hydrogen peroxide elimination (*in vitro*)	Pellavio et al., 2017
	Hesperetin	Microglial AQP4 ↓	Amelioration of diabetic retinal edema (rat model)	Kumar et al., 2014
	Alpinetin	Lung endothelium AQP1 ↑	Amelioration of SAP-induced acute lung injury (*in vitro*)	Liang et al., 2016
	Naringenin	Mucosal epithelial cells of colon AQP3 ↑	Amelioration of intestinal water absorption	Yin et al., 2018
		HeLa cells AQP(ND)	Hydrogen peroxide elimination (*in vitro*)	Pellavio et al., 2017
	Liquiritigenin	Kidney AQP4 ↓	Reduction of kidney inflammation (rat model)	Hongyan et al., 2016
	Epigallocathechin Gallate	Salivary gland AQP5 ↑	Amelioration of xerostomia in Sjögren syndrome (mouse model)	Saito et al., 2015
		SKOV3 AQP5 ↓ (cancer cell line)	Inhibition of ovarian tumor growth (*in vitro*)	Yan et al., 2012
		Spinal cord AQP4 ↓	Reduced edema in acute SCI (rat model)	Ge et al., 2013
Chalcones	Phloretin	AQP9 (inhibition)	Anti-inflammatory and anti-oxidative action (*in vitro*)	Matsushima et al., 2014
Stilbenes	Resveratrol	Human keratinocyte AQP3 ↓	Inhibition of keratinocyte proliferation (*in vitro*)	Wu et al., 2014
		Brain AQP4 ↓	Amelioration of cerebral I/R injury (rat model)	Li et al., 2015
Isoflavonoids	Genistein and Daidzein	Uterine AQP1 ↑	Uterine responsiveness to estrogens (rat model)	Möller et al., 2010
	Puerarin	Brain AQP4 ↓	Reduction of brain damage and inflammation	Wang et al., 2018

*Arrows: ↑, upregulation; ↓, downregulation; SCI, spinal cord injury; ND, specific AQP homolog to be defined*.

### Curcuminoids

Curcuminoids (or curcumins) are characterized by a pronounced yellow color composed of linear diarylheptanoids. They are represented by curcumin and its derivatives (i.e., demethoxycurcumin and bisdemethoxycurcumin). Curcuminoids have been tested in particular for their anti-oxidant activity.

*Curcumin* is a non-flavonoid polyphenol isolated from spice turmeric, and known for playing anti-inflammatory, antioxidant, anti-proliferative, and anti-angiogenic activities (Tsao, 2010). The beneficial effects of curcumin on human health, however, are downplayed by its poor absorption and bioavailability (Anand et al., 2007). Liposomal curcumin or curcumin nanoparticles have increased bioavailability, while structural analogs of curcumin such as EF-24 feature higher stability and faster absorption (Santiago-Vázquez et al., 2016). EF-24 has been demonstrated effective against cancer (Yang et al., 2014; Santiago-Vázquez et al., 2016), Parkinson's (PD), and Alzheimer's (AD) diseases (Pal et al., 2011).

The central nervous system expresses various AQPs (Badaut et al., 2014) and studies using rodent brain have suggested important roles for AQP1, AQP4, and AQP9 in controlling water transport and volume homeostasis (Badaut et al., 2014). AQP1 is highly expressed in choroid plexus, a secretory epithelium which plays a role in cerebrospinal fluid (CSF) formation and secretion (Nabiuni et al., 2013). After brain injury, mice deficient for AQP1 displayed a decreased intracranial pressure and improved survival compared to wild type mice (Oshio et al., 2005). This strongly suggests that AQP1 downregulation might be protective against several neurological disorders characterized by increased intracranial pressure. Notably, curcumin decreases, in a dose-dependent manner, the AQP1 level in choroidal epithelial cells isolated from the lateral ventricle of Wistar rats (Nabiuni et al., 2013). Curcumin could also act inhibiting choroid plexus AQP1 since in a study using Hela cells this phytocompound has been recently suggested to have a direct gating action on some AQPs (Pellavio et al., 2017). AQP4, the most characterized brain water channel, is located mainly on astrocytic endfeets that are in strict contact with blood vessels (Badaut et al., 2014). Curcumin counteracts the brain edema and the effect might include the modulation of the expression of various AQPs, especially AQP4. Indeed, current medical and surgical therapies available for the treatment of intracerebral hemorrhage (ICH) do not adequately control brain edema. Interestingly, curcumin dose-dependently reduced both the gene expression and protein abundance of AQP4 and AQP9 but not those of AQP1 in a mouse model of ICH (Wang et al., 2015). Likely, the protective effects of curcumin may also involve the downregulation of specific water channels.

In a rat model of hypoxic-ischemic brain injury, curcumin significantly reduced the brain edema, and the effect was associated with a relevant morphological amelioration of the damage at the blood-brain barrier, increase of NOS activity and AQP4 expression (Yu et al., 2012). Similarly, in a rat model of hypoxia-hypercapnia, curcumin injection attenuated brain edema and restored the levels of AQP4 expression (Yu et al., 2016b). In the mice model of traumatic brain injury, pre- or post-treatment with curcumin reduced the cerebral edema, the pericontusional expression of IL-1β, and reversed the induction of AQP4 (Zhang et al., 2014). The beneficial effect of curcumin was also observed in an animal model of SCI (impaired motor function and spinal cord edema); curcumin counteracted the abnormal activation of JAK/STAT signaling pathways and reduced the glial fibrillary acidic protein (GFAP) and AQP4 overexpression (Zu et al., 2014). While most available data highlight the beneficial effects of curcumin in neurologic diseases, curcumin could aggravate some CNS manifestations in experimental lupus erythematous. Indeed, curcumin treatment was associated with increased cerebral water content and AQP4 expression in mice with systemic lupus erythematous, likely depending on worsened cerebral atrophy and astrocytosis (Foxley et al., 2013). All together the above-mentioned results suggest that AQP4 is the main target through which curcumin would exert its action on brain edema and SCI.

In cultured human ovarian CaOV3 cells, stimulation with the endothelial growth factor (EGF) promoted AQP3 expression and CaOV3 cell migration. AQP3 knocking down by siRNA was associated with significant impairment of CaOV3 cell migration. Also in this context, curcumin (or a stable analog) proposed for therapeutic treatment of ovarian cancer (Terlikowska et al., 2014), downregulated AQP3 and reduced cell migration in CaOV3, an effect mediated by inhibition of EGFR signaling (Ji et al., 2008).

As shown for quercetin and naringenin (see below), in HeLa cells curcumin elicited antioxidant effects by reducing the hydrogen peroxide cellular content, probably by decreasing its entry into the cell through AQPs-mediated mechanisms (Pellavio et al., 2017). Modulation of AQPs by curcumin in cancer cells resulting in elimination of hydrogen peroxide may not always result beneficial to health when considering that accumulation of hydrogen peroxide (among other ROS) is a mechanism by which a number of conventional anti-cancer treatments govern cancer cells to death.

Curcumin could also influence various physiological functions through interactions with different ion channels and transporters involving several signaling pathways, from the well-known CFTR to voltage-gated potassium channels, volume-regulated anion channel (VRAC), Ca^2+^ release-activated Ca^2+^ channel (CRAC), and glucose transporters (Zhang et al., 2014). More research is needed in this respect, to better highlight the role of curcumin as modulator of various channel functions and pointing to its protective effects in various disease. Moreover, the use of curcumin could be useful also for research purposes since it could help the understanding of the interplay between AQPs system and other membrane transporters (Zhang et al., 2014).

### Flavonoids

Flavonoids are the biggest representative subgroup of polyphenols, with more than 4,000 molecules described (Harborne and Williams, 2000; Cheynier, 2005).

*Pinocembrin* is a natural flavonoid compound, which has been isolated from several plants, such as ginger roots and wild marjoram, honey, and propolis (Massaro et al., 2014; Lan et al., 2016). Pinocembrin exerts pleiotropic effects: reduces reactive oxygen species (ROS) production, apoptosis, and controls mitochondrial functions (Massaro et al., 2014). *In vitro* evidence revealed that pinocembrin can cross the blood-brain-barrier passively indicating possible therapeutic use in nervous system diseases (Yang et al., 2012). In an animal model of focal cerebral ischemia induced by middle cerebral artery occlusion (MCAO), several inflammatory cytokines, tumor necrosis factor-α (TNF-α), and interleukin-1β (IL-1β), inducible nitric synthase (iNOS) and AQP4 were significantly upregulated in the ischemic brain (Gao et al., 2010). Administration of pinocembrin *via* tail vein injection ameliorated the neuronal apoptosis and the edema as well as the typical alterations of endothelial cells and capillaries characterizing ischemia. Pinocembrin treatment decreased the production of cytokines and the expression iNOS and AQP4. These findings suggest that the protective response triggered by the flavonoid might be due to reduced inflammatory signals and decreased level of AQP4, which has been associated with edema subsequent to cerebral ischemia (Gao et al., 2010).

*Chrysin* is a natural flavonoid occurring in honey and propolis, various fruits, vegetables and mushrooms (Nabavi et al., 2015). Chrysin displays fundamental biological anticancer actions reducing cell proliferation and promoting apoptosis, especially in leukemia cells (Monasterio et al., 2004). In animal studies, UVB and UVA radiation induced skin dehydration and this step was associated with decrease of the expression level of AQP3, one of the main skin AQPs. Other changes included ROS release and apoptosis (Wu et al., 2011). Topical application of chrysin improved the UV-induced skin damage, and significantly increased keratinocyte AQP3, suggesting a chrysin-mediated protection of the deleterious effects exerted by UVs on human skin.

*Quercetin* is one of the most abundant bioflavonoids in the human diet. It is largely present in different vegetables including onions and broccoli, fruits such as apples, berry crops, and grapes. Quercetin is also found in some herbs, tea, and wine. Similarly to other polyphenols, quercetin displays pleiotropic properties as antioxidant and anti-inflammatory compound. Plant extract of quercetin is the principal ingredient of many potential anti-allergic drugs, supplements, and enriched products, being highly competent in inhibiting IL-8 action, suppressing IL-6 and intracellular calcium level increase (Mlcek et al., 2016). Retinas from streptozotocin-induced diabetic rats showed a remarkable increase of pro-inflammatory cytokines such as TNF-α and IL-1β and a considerable augmentation of AQP4 level, which could mediate the movement of water underlying the retinal edema (Kumar et al., 2014). Changes were accompanied with a significant reduction of retinal glutathione (GSH) and antioxidant enzymes [superoxide dismutase (SOD) and catalase (CAT)]. Oral administration of quercetin leads to an important neuroprotective effect which was characterized by impaired inflammatory cytokines production and release, restoration of GSH, SOD, and CAT levels and significant reduction of AQP4 in Müller cell endfeet and perivascular space with consequent decrease of the edema affecting the retina of diabetic rats. The observed amelioration of retinal edema was suggested as due to quercetin-dependent AQP4 downregulation on Müller cell endfeet and perivascular space. Another orthodox AQP water channel, AQP5, was found to be involved in the beneficial action of quercetin in attenuating the damaged salivary secretion induced in a murine model of impaired salivation by radiation exposure (Takahashi et al., 2015). Quercetin upregulated AQP5 expression and calcium uptake and suppressed the oxidative stress and inflammatory responses induced by the radiation exposure. Increased AQP5 levels were also observed in a mouse model of influenza A virus (IAV)-induced lung injury where AQP5 lung increased after treatment with flavonoid extracts from the Lamiaceae plant *Mosla scabra* (Yu et al., 2016a). The lung AQP5 modulation by the flavonoid was interpreted as a way to restore the normal water permeability alleviating the IAV-induced pulmonary inflammation and apoptosis.

AQP3, 5, 8, and 9 have been reported to facilitate the transmembrane diffusion of hydrogen peroxide in mammalian cells (Almasalmeh et al., 2014; Rodrigues et al., 2016; Watanabe et al., 2016). This is an important aspect since cellular oxidative stress can interfere with water permeability. In HeLa cells, the role of AQPs as target of antioxidant compounds acting on the osmotic water diffusion in the presence of oxidative stress condition has recently been studied (Pellavio et al., 2017). Quercetin appeared to modulate water transport and acted as antioxidant by increasing the expression of AQP3 and AQP8 (together with that of AQP1 and AQP11) at both mRNA and protein level. Particularly, with quercetin, the water permeability decrease caused by oxidative stress was prevented or restored. Furthermore, quercetin significantly reduced the hydrogen peroxide content to levels even lower than those of control cells. Thus, regulation of AQPs gating by antioxidant compounds like quercetin could represent a novel mechanism to modulate exogenously cell signaling and survival during stress, acting on key signaling pathways in cancer and degenerative diseases (see Tamma et al., 2018 for a review).

*Hesperetin* is a flavanone isolated from several fruits and highly expressed in the *Citrus* species. This flavonoid has different biological properties, including antioxidant, anti-inflammatory, and anticancer effects (Ahmadi et al., 2015; Bodduluru et al., 2015). Its beneficial actions have been showed in different organs including liver, heart, and kidney (Roohbakhsh et al., 2015; Miler et al., 2016). Neuroprotective effects have also been reported, in particular in the treatment of diabetic retinopathy where inflammation, oxidative stress, and neurovascular disorders are involved. Streptozotocin-induced diabetic rats receiving hesperetin for 24 weeks showed restoration of retinal levels of GSH associated to a positive modulation of antioxidant enzyme activities. An inhibition of caspase-3 activity and expression of GFAP was also seen along with reduced inflammatory cytokines (Kumar et al., 2013). Treatment with hesperetin was accompanied by downregulation of AQP4 at Müller cell endfeet and consequent reduction of the edematous state indicating modulation of AQP-associated water permeability.

*Alpinetin* is a flavanone isolated from the seed of *Alpinia katsumadai* (Zingiberaceae). The compound is widely used in Korean traditional medicine (Lee et al., 2012). Alpinetin has been found to control cell signaling pathways at the base of cell growth, proliferation, and apoptosis (Wang et al., 2013). Alpinetin also causes vasorelaxation (Wang et al., 2001) and counteracts the hydrogen peroxide-induced vascular smooth muscle cell proliferation and migration (Li and Du, 2004). In lipopolysaccharide (LPS)-induced lung injury, alpinetin prevented the LPS-induced TNF-α, IL-6, and IL-1β release and alleviated the inflammatory associated lung hystopathological alterations (Hou et al., 2009). Considerable AQP1 downregulation, a condition negatively correlated with pulmonary edema, has been described in acute lung injury (ALI) and, associated with severe acute pancreatitis (SAP). Interestingly, alpinetin has been found to inhibit TNF-α expression, promote human pulmonary microvascular endothelial cell proliferation and increase the expression level of AQP1 thereby improving the SAP-induced ALI symptoms (Liang et al., 2016). Overall, these findings propose alpinetin as possible therapeutical tool against lung inflammation diseases.

*Naringenin* is a natural flavonoid widely found in citrus fruits and tomatoes, that has been reported to act as anti-inflammatory, anti-atherogenic, anti-mutagenic, hepatoprotective, and anticancer agent (Yin et al., 2018). Naringenin relieved the loperamide-induced constipation by targeting AQP3. It is known that AQPs are primarily expressed in the mucosal epithelial cells in the colon, in which AQP3 plays a central role in water reabsorption across colonic surface cells. Yin et al. found that naringenin increased the mRNA and protein expression levels of AQP3 in the colon, both in apical and lateral mucosal epithelial cells. Furthermore, a positive correlation was observed between this increase in the AQP3 level and the increase in fecal water content (Yin et al., 2018). As also shown for quercetin, the antioxidant effects elicited by naringenin in HeLa cells have been recently related to its capacity to facilitate hydrogen peroxide elimination through AQPs. These observations would strengthen the role of AQPs as physiologic modulators of hydrogen peroxide diffusion in mammalian cells (Pellavio et al., 2017).

*Liquiritigenin* belongs to the chiral flavanone family and is an important compound extracted from *Glycyrrhiza uralensis* and found in a variety of plants, including *Glycyrrhiza glabra* (licorice). Liquiritigenin possesses kinds of healthy properties including antioxidation, anti-inflammation, antidiabetes, cardioprotection, and neuroprotection (Hosseinzadeh and Nassiri-Asl, 2015). As regarding the mechanisms, the inhibition of NF-κB and MAPK signaling pathways would be at basis of its antioxidant effects. Liquiritigenin has been shown to exhibit renal protective effects in the animal model of potassium oxonate-induced hyperuricemia, as well, by targeting the AQPs (Hongyan et al., 2016). AQP4 would play an important role in inflammatory responses involving the kidney, also by the regulation of endoplasmic reticulum stress. In the hyperuricemic rat, liquiritigenin has been able to suppress the activation of renal AQP4/NF-κB/IκBα signaling and nod-like receptor protein 3 (NLRP3) inflammasome, resulting in renal inflammation reduction. These findings would suggest that liquiritigenin could act as a potential drug for the treatment of hyperuricemia and renal injury by targeting the AQPs system.

*Epigallocatechin gallate* (EGCG) is a flavonol esterified with gallic acid mainly found in the green tea *Camellia sinensis* L. EGCG accounts for more than 50% of total green tea polyphenols. This phenolic compound and/or its metabolites exert cardioprotection, neuroprotection, renal protection, osteoprotection, and anticancer actions. Beneficial effects have also been shown in diseases with metabolic disorders such as obesity, metabolic syndrome and type 2 diabetes (Afzal et al., 2015). At a molecular level, EGCG promotes the expression and the activity of several anti-oxidant and anti-inflammatory enzymes. EGCG is also proved to counteract the activation of Toll-like receptor 4 (TLR4) (Marinovic et al., 2015) and nuclear factor-κB (NF-κB) (Albuquerque et al., 2016), pathways associated with the production of inflammatory cytokines. In a mouse model of Sjögren syndrome, treatment with EGCG increased the abundance of AQP5 at the apical plasma membrane of the acinar cells. AQP5 expression resulted upregulated by mechanisms leading to protein kinase A (PKA) activation and NF-κB inhibition (Saito et al., 2015). Conversely, by means of other pathways, EGCG was found to downregulate the expression of AQP5 in the ovarian cancer cell line SKOV3. The lower abundance of AQP5 was suggested to counteract the tumor growth through NF-κB activation (Yan et al., 2012). Considerable decrease of the spinal cord water content was seen in a work employing a rat model of acute SCI where EGCG was administered immediately following the injury (Ge et al., 2013). The anti-edema action exerted by EGCG was ascribed to the marked reduction of spinal cord AQP4 induced by the EGCG administration.

### Chalcones

Chalcones are a variety of aromatic ketones, precursors of flavonoids, and isoflavonoids. They are abundant in edible plants and their derivatives have been reported to have an extremely wide variety of biological activities (i.e., anti-bacterial, anti-fungal, anti-neoplastic, anti-inflammatory, anti-diabetic, anti-obesity, immunosuppressant actions) depending on the substitution made on them (Mahapatra et al., 2015).

*Phloridzin*, one of the most characterized bioactive chalcones, is also a competitive inhibitor of the isoforms 1 and 2 of the sodium glucose cotransporter (SGLT1 and SGLT2, respectively) as it competes with D-glucose in binding the carrier. This action leads to a decrease in glucose absorption and reabsorption by the small intestine and renal proximal tubules, respectively, lowering the glucose level in the blood. However, phloridzin is not an effective drug because when orally consumed, it is nearly entirely converted into phloretin and glucose by hydrolytic enzymes in the small intestine.

*Phloretin*, an abundant chalcone in apples, is a protective agent with anti-oxidative stress and anti-inflammatory actions (Aliomrani et al., 2016). Phloretin is also known for gating the aquaglyceroporins by inhibiting the AQP-mediated transport of glycerol and urea (Tsukaguchi et al., 1999; Calamita et al., 2008, 2012; Rodriguez et al., 2014), a metabolic intermediate substrate of gluconeogenesis and triacylglycerols (TAG) synthesis (Calamita et al., 2012), and a variety of urea transporters and urea-conducting AQPs such as AQP3, AQP7, and AQP9 (Shayakul et al., 2001; Fenton et al., 2004). AQP9-facilitated urea extrusion out of liver was evoked to explain the loss of urea and diuresis that characterizes mice submitted to high protein diet. This function played by liver AQP9 can be blocked by phloretin (Jelen et al., 2012). It is tempting to think that the anti-inflammatory action exerted by phloretin may also involve the inhibition of AQP9, an AQP that has been suggested to play a role in inflammation (Matsushima et al., 2014).

### Stilbenes

Stilbenes belong to the family of phenylpropanoids and are better known as stilbenoids, their hydroxylated derivatives. The most studied stilbenoid is resveratrol, a compound having numerous health benefits.

*Trans-resveratrol* is a natural hydroxystilbene found in a variety of edible plants including grapes, blueberry, raspberry, and senna leaves. Research on the biological actions of resveratrol on human health has focused on cancer, neurodegenerative, and cardiovascular diseases, and metabolic disorders. At molecular level, resveratrol plays pleiotropic effects including inhibition of kinases, anti-inflammatory, analgesic and anti-cancer activities, and detoxification, by inhibiting the aryl hydrocarbon and dioxin receptor (AhR), (de Medina et al., 2005). AhR stimulation causes oxidative stress, inflammation, apoptosis and immunosuppression, and is associated with an increased risk of osteoporosis, cancers and metabolic disorders such as diabetes (Zollner et al., 2010). As a polyphenol, resveratrol can also act as tyrosine kinases inhibitor and as a modulator of the mitogen activated protein kinase/extracellular signal-regulated kinase 1/2 (MEK-ERK1/2), mitogen-activated protein kinases (MAPK), activator protein 1 (AP-1), and NF-κB pathways in different tissues (Yu et al., 2001; Gao et al., 2004; Kundu et al., 2004). Resveratrol has been reported to influence the gene expression of various AQPs (see Cataldo et al., 2017 for a review). In human keratinocytes, AQP3 has been found to play a pivotal role in skin hydration, although overexpression of AQP3 is also linked with hyperplastic epidermal disorders (Nakahigashi et al., 2011). In normal human epidermal keratinocytes (NHEKs), treatment with resveratrol reduced cell proliferation and the expression of AQP3, the major skin AQP. Particularly, AQP3 downregulation appeared to be secondary to ERK signaling inhibition via upregulation of both Sirtuin 1 (SIRT1) and aryl hydrocarbon receptor AhR (Wu et al., 2014). These novel findings may be important to drug design and development against hyperproliferative skin disorders.

Cerebral ischemia-reperfusion (I/R) is associated with a strong increase of ROS production and brain edema. Resveratrol exerts a beneficial action by modulating the activity of SOD and reducing the iNOS and AQP4 expression levels (Li et al., 2015). More recently, functionalized AQP4 antibody nanoparticles were synthesized to deliver resveratrol in rat optic nerve transection. These engineering nanoparticles displayed high efficacy in reducing oxidative damage and AQP4 immunoreactivity thus preserving the visual function (Lozić et al., 2016).

### Isoflavonoids

The isoflavonoids genistein and daidzein are important components of *Leguminosae*. Based on their chemical structure they function as phytoestrogens displaying anti-tumor features. These phytocompounds play an important role in modulating the genes involved in controlling cell-cycle progression.

*Genistein* and *daidzein* are considered the “sharp edge of balance” between cell survival and cell progression because they control the activation or the inhibition of pivotal signal molecules such as NF-κB. Importantly, they also play a role in reversion of epigenetic events associated with prostate cancer (Adjakly et al., 2013). The bioavailability of these compounds is strongly influenced by gut microbiota, antibiotic administration and individual's age and health status (Franke et al., 2014). A work addressing the effects of lifelong dietary isoflavone on estrogen sensitive tissues was carried out studying the effects of genistein and daidzein on rat uterus (Möller et al., 2010). The effect of genistein either alone or in association with daidzein was compared to that of isoflavone-free diet in rats throughout their whole lifetime. The dietary isoflavone pre-exposure resulted in a much stronger uterine weight increase following external ERα-mediated estrogenic stimuli than that seen in the phytoestrogen–free group. Gene expression analysis showed that the uterine levels of AQP1, and, at a lesser extent, those of AQP3, AQP5, and AQP9, were increased by ovary estrogens. This modulation was considerably influenced by the isoflavone-containing diets, likely by an epigenetic mechanism. Lifelong dietary isoflavone ingestion was suggested to increase the uterine responsiveness to ERα-mediated estrogenic stimuli in female rats where the water homeostasis was highly affected whereas the proliferation rate remained unchanged.

*Puerarin* is a flavonoid glycoside that is extracted from the root of the leguminous plants *Pueraria lobata* and Thomson Kudzuvine Root. Puerarin displays a series of beneficial activities on hangover, cardiovascular disease, osteoporosis, neurological dysfunction (ischemic stroke, cerebrovascular disease) fever, and liver injury both in clinical treatment and experimental research (Wang et al., 2018). In addition to inhibit inflammation, protective effects elicited by puerarin against cerebral damage would be related to modulation of AQP4 function. Hence, AQP4, which can be mainly found in the primate and rodent perivascular astrocyte end feet, would play a role not only in water movement, but also, as previously reported, in neuroinflammation and brain edema. AQP4 expression would be increased by pro-inflammatory factors like TNF-α. Furthermore, increased phosphorylation of MAPK (p38, ERK1/2, c-Jun N-terminal kinase 1/2) would participate in AQP4 regulation in astrocytes exposed to the inflammatory cytokines released by microglia under the condition of hypoxia, too. Various interventions have been performed to prevent or to treat people rapidly ascending to high altitude. Currently, traditional Chinese medicine has been used to prevent or treat symptoms although the specific mechanisms are still a matter of debate. In rats undergone hypobaric hypoxia, puerarin was able to elicit protective effects against cerebral edema by inhibiting the increase of AQP4 through inhibition of TNF-α release and by counteracting the activation of NF-κB and MAPK pathway (Wang et al., 2018).

## Capsaicinoids

*Capsaicin* is the most representative compound of a group of phytochemicals called capsaicinoids also including dihydrocapsaicin, nordihydrocapsaicin, and some other compounds such capsinoids. In chili peppers, capsaicin, a phenolic amide, gives the sensation of spiciness by acting through the transient receptor potential vanilloid-1 (TRPV1). TRPV1 is a non-selective permeable cation channel expressed in brain, bladder, kidneys, intestines, keratinocytes of epidermis, glial cells, liver, and polymorphonuclear granulocytes, mast cells, and macrophages (Reyes-Escogido Mde et al., 2011). In tumor cells, capsaicin inhibits cell growth and promotes apoptosis by increasing the intracellular calcium concentration and ROS levels, disrupting mitochondrial membrane transition potential and activating NF-κβ transcription factor (Chapa-Oliver and Mejia-Teniente, 2016). In addition, capsaicin stimulates the phosphorylation of p53 at serine-15 and its acetylation through downregulation of SIRT1. Altogether, these posttranslational modifications lead to apoptosis (Ito et al., 2004). Similarly to anti-obesity, anti-diabetic, and anti-inflammatory compounds, capsaicin plays multiple roles in nociceptive heat sensation. Capsaicin can activate sympathetic system to induce brown adipose tissue thermogenesis (Saito, 2015).

Capsaicin upregulated TRPV1 and AQP5 in rabbit salivary glands (Table 2). Specifically, in transplanted rabbit submandibular gland cells, capsaicin upregulated and stimulated the translocation of AQP5 from an intracellular pool to the plasma membrane via TRPV1 signaling and ERK phosphorylation (Ding et al., 2010; Zhang et al., 2010). This finding may provide a new therapeutic tool to stimulate submandibular gland hypofunction.

**Table 2:** Modulation of AQPs by non-polyphenolic phytocompounds and related beneficial effects.

**Phytochemical**	**Functional derivative**	**Modulated AQP**	**Beneficial effect**	**References**
Capsaicinoids	Capsaicin	Submandibular salivary gland AQP5 ↑	Amelioration of submandibular salivary gland hypofunction (rabbit model)	Ding et al., 2010; Zhang et al., 2010
Monoterpenoids	Carvacrol	Brain AQP4 ↓	Reduction of ICH-induced brain edema (mouse model)	Zhong et al., 2013
	Marrubin	HeLa cells AQP(ND)	Hydrogen peroxide elimination (*in vitro*)	Pellavio et al., 2017
Triterpenes	Bacopasides I and II	AQP1 (inhibition)	Reduction of cancer cell migration (*in vitro*)	Pei et al., 2016
	18β-glycyrrhetinic acid (β-GA)	Nasal mucosa AQP1 ↓	Treatment against allergic rhinitis (rat model)	Li et al., 2015
	Glycyrrhizic acid	Renal AQP2 ↓	Protection against renal failure (rat model)	Sohn et al., 2003
	Ginsenoside Rg1	Brain AQP4 ↓	Protection against brain ischemia (rat model)	Zhou et al., 2014
	Ginsenodise Rg3	AQP1 ↓	Anti-metastatic effect (*in vitro*)	Pan et al., 2012
Isothiocyanates	Sulphoraphane	AQP4 ↑	Reduction of brain edema (rat model)	Zhao et al., 2005
Tetrahydroanthracene	(R)-Aloesaponol III 8 methyl ether	HeLa cells AQP(ND)	Hydrogen peroxide elimination (*in vitro*)	Pellavio et al., 2017

*Arrows: ↑, upregulation; ↓, downregulation; ND, specific AQP homolog to be defined*.

## Terpenes

Terpenes are a large and diverse class of organic compounds derived biosynthetically from units of isoprene. They are produced by a variety of plants and even some insects. Terpenoids are compounds related to terpenes characterized by an isoprenoid chemical structure as they may include some oxygen functionality or some rearrangement. Terpenoids are present in a great variety of fruits, vegetables and medicinal plants, representing the largest and most diverse class of chemicals among the myriad of compounds produced by plants. Terpenoids are used extensively for their aromatic qualities and play a role in traditional herbal remedies. The terms terpenes and terpenoids are often used interchangeably. As many other bioactive phytocompounds, terpenes, and terpenoids have been reported to influence the expression of AQPs (Table 2).

### Monoterpenoids

*Carvacrol* (or cymophenol) is a monoterpenoid extracted from many plants of the *Lamiaceae* family. Carvacrol has been reported to exert neuroprotective effects in central nervous system diseases such as AD and cerebral ischemia (Zhong et al., 2013). Modulation of brain AQP4 was reported in a preclinical study employing a bacterial collagenase-induced ICH murine model to address the effect of the monoterpenoid on cerebral edema after ICH. Administration of carvacrol improved the neurological deficits following ICH by significantly reducing cerebral edema and AQP4 in perihematomal area (Zhong et al., 2013). It was suggested that carvacrol exerts its protective effect on ICH injury by ameliorating AQP4-mediated cerebral edema.

*Marrubiin* (MARR) is a terpenoid abundantly found in many Lamiaceae species (i.e., *Marrubium vulgare, Plomis bracteosa, Leonotis nepetifolia*). MARR is a compound featuring high stability and limited catabolism, two properties favoring its bioavailability: MARR is reported to exert cardioprotective, vasorelaxant, gastroprotective, and antidiabetic effects (Popoola et al., 2013). Antioxidant action involving AQPs has been reported in a recent work where pre- or post-treatment of heat-stressed HeLa cells with MARR prevented or reversed, respectively, the intracellular H_2_O_2_ accumulation induced by the heat (Pellavio et al., 2017).

### Triterpenes

*Bacopasides* are triterpene saponins isolated from the medicinal plant *Bacopa monnieri*. Recently, two structurally related bacopaside compounds, bacopaside I and bacopaside II, have been shown to block differentially the transport activity of AQP1, a finding that fitted with predictions made by *in silico* molecular modeling (Pei et al., 2016). When tested in migration assays using HT29 and SW480 cells, two colon cancer cell lines characterized by high and low expression levels of AQP1, respectively, both bacopasides impaired migration of HT29 cells showing minimal effect on migration of SW480 cells. Based on these results bacopasides were suggested as possible novel lead compounds for pharmaceutical development of selective AQPs blockers in cancer treatment.

The triterpenoid *18β-glycyrrhetinic acid* (β-GA) is a natural compound derived from *Glycyrrhiza glabra* (licorice) root shown to exert antiviral, antitumor and immunosuppressive effects. Studies using rat models of nasal mucosa of allergic rhinitis (AR) showed that intranasal administration of β-GA downregulates AQP1 together with that of eotaxin 1 (CCL11) and eosinophil (EOS) in nasal mucosa of allergic rhinitis rats and cast effects on inhibiting the progress of AR (Li et al., 2015).

*Glycyrrhizic acid*, a triterpenoid also known as glycyrrhizin, is the main sweet-tasting constituent of licorice root. Based on its inhibiting effect on liver cell injury glycyrrhizin is used for the treatment of chronic viral hepatitis and cirrhosis (van Rossum et al., 1998). Glycyrrhizin is also employed to prevent disease progression in subjects with acute onset autoimmune hepatitis (Yasui et al., 2011). Enoxolone, the aglycone of the glycyrrhizic acid, is used to prevent liver carcinogenesis in patients with chronic hepatitis C. Glycyrrhizin was reported to influence the renal functions by modulating AQP2 and AQP3, two AQPs expressed in the principal cells of the renal distal tubules and collecting ducts where they mediate the antidiuretic hormone (ADH)-induced water reabsorption (Kang et al., 2003). Using a rat model of gentamicin-induced acute renal failure the protective effects of glycyrrhizin downregulated AQP2 in the inner and outer renal medulla, and cortex (Sohn et al., 2003) suggesting that this triterpenoid involves renal AQPs in its beneficial action on renal function.

*Ginsenoside Rg1* (Rg1) is a triterpenoid saponin known as one of the main compounds harvested from ginseng with pharmaceutical action and potential neuroprotective properties, empirically used in traditional Chinese medicine to treat stroke (Xie et al., 2015). A study using a rat model of cerebral ischemia/reperfusion showed that the neuroprotective effect of Rg1 against blood-brain-barrier disruption involves downregulation of brain AQP4 expression (Zhou et al., 2014).

*Ginsenoside Rg3* (Rg3), a triterpene saponin, is one of the bioactive extracts contained in ginseng root. Rg3 has been shown to have anticancer activity in various cancer models. Thus, in the highly metastatic prostate cancer cell line PC-3M, treatment with Rg3 was found to lead to a remarkable inhibition of cell migration. In particular, exposure of PC-3M cells to Rg3 suppressed the expression of AQP1, an AQP with a role in cell migration. The anti-metastatic effect of Rg3 was found to occur through the p38 MAPK pathway and some transcription factors acting on the *Aqp1* gene promoter (Pan et al., 2012).

## Sulphoraphane

*Sulphoraphane* (SUL) is a chemical belonging to isothiocyanates, a group of organosulfur compounds. SUL was identified in broccoli sprouts, which, of the cruciferous vegetables have the highest concentration of this compound (Zhang et al., 1992). Among the reported beneficial effects sulforaphane appears to be a promising phytochemical with neuroprotective properties (Tarozzi et al., 2013).

Using a rodent controlled cortical impact injury model of traumatic brain edema it was observed that post-injury administration of SUL counteracted AQP4 loss in the contusion core and further increased the protein levels of this water channel in the penumbra region compared with injured animals receiving vehicle. This increase in AQP4 expression was accompanied by a significant amelioration of cerebral edema (Zhao et al., 2005). It was suggested that the reduction of the edema in response to SUL administration could be ascribed to water clearance through AQP4 from the injured brain.

## Tetrahydroanthracenes

Tetrahydroanthracenes are polycyclic aromatic phytocompounds deriving from the phytochemical hydrogenation of anthracene in *Liliaceae*. This reaction is influenced by light exposition in roots of *Aloe* plants and tetrahydroanthracenes are markers of subterranean anthranoid metabolism. These molecules are used mainly to treat parasitic infections with potential anti-oxidant properties because of their chemical structure.

(***R***)-*Aloesaponol III 8-methyl ether*. The (***R***)-Aloesaponol III 8-methyl ether (ASME) was extracted for the first time from *Eremurus persicus* root but it is contained in other plants such as *Aloe saponaria, Kniphonia foliosa, Eremurus chinensis*, and others. ASME is a tetrahydroanthracene known for having biological activity against *Leishmania* infection. Like the terpenoid MARR, ASME improved the HeLa cells membrane permeability by restoring the AQP-mediated cellular extrusion of H_2_O_2_ contributing to the reduction in oxidative stress (Pellavio et al., 2017). However, further work is needed to better investigate this preliminary observation since the specific AQP channel responsible for the ASME-induced efflux of H_2_O_2_ out of the cells is still elusive.

## Bioactive extracts

The beneficial effects of a number of plants are likely due to their original composition and the synergistic action played by the different bioactive phytochemicals they contain rather than to a unique active compound. Here, we review bioactive extracts from plants whose healthy effects have been reported to involve modulation of AQPs (Table 3).

**Table 3:** Modulation of AQPs by bioactive extracts and related beneficial effects.

**Bioactive extract**	**Modulated AQP**	**Beneficial effect**	**References**
Extracts of *Heliotropium indicum*	Eye AQP0 ↑	Alleviation of cataract (rat model)	Kyei et al., 2015
Extracts of aged garlic (S-allylmercapto-l-cysteine)	SPC-A1 cell line AQP5 ↑	Anti-inflammatory properties in COPD	Yang et al., 2016
Extracts of Chinese herbs (Isotetrandrine)	Eye AQP4	Amelioration of auto-immune disorders in NMO	Sun et al., 2016

*Arrow: ↑, upregulation*.

*Heliotropium indicum* is a plant belonging to family of *Boraginaceae* used in folk medicine in many countries. In Ghana, extracts of *H. indicum* are employed as a remedy against cataract formation without any scientific evidence. The extract of this plant contains an original composition showing numerous bioactive compounds with antibacterial, antitumor, anti-inflammatory and diuretic activities. In a rat model of selenite-induced cataract development, the aqueous total extract of *H. indicum* significantly alleviated cataract at all dose levels tested (0.1–3.0 mg/ml) preserving AQP0, an AQP highly expressed in fiber cells of lenses, and other markers of lens transparency proteins (Kyei et al., 2015).

Garlic has shown versatile therapeutic action in the prevention and treatment of pathologies such as chronic obstructive pulmonary disease (COPD). However, the specific garlic bioactive component underlying this medicinal activity remains elusive. Viscous COPD mucus secretions have been explained also due to a down-regulation to which AQP5, an AQP water channel highly expressed in lungs, undergoes. Interestingly, *S-allylmercapto-l-cysteine* (SAMC), one of the bioactive phytochemicals found in aged garlic, improved the LPS-induced mucus secretion of a COPD cell model, the human airway submucosal gland cell line (SPC-A1), up-regulating AQP5 and mucin 5AC (MUC5AC) *via* NF-κB signaling pathway (Yang et al., 2016).

*Isotetrandrine* is a biscoclaurine alkaloid, a bioactive compound isolated from traditional Chinese herbs known for reducing the astrocyte cytotoxicity in the neurological autoimmune disorder such as neuromyelitis optica (NMO). This phytochemical was reported to be a small molecule inhibitor of NMO-IgG binding to AQP4 without impairing the expression and water channel activity of AQP4 (Sun et al., 2016).

## Conclusions and future perspectives

Foods are enriched with myriads of bioactive phytocompounds, and research on their effects on human health is a quickly growing field. The recent recognition that AQPs are among the targets of food phytochemicals paves the way to potentially novel therapeutic options dealing with prognosis and cure of highly prevalent diseases (i.e., metabolic disorders, cancer, neurological diseases, and inflammatory chronic diseases). While the molecular mechanisms by which phytocompounds modulate the expression or gating (i.e., by phloretin, curcumin, and bacopaside II) of AQPs remains to be fully ascertained, there are no doubts that the list of functional food ingredients and herbal phytochemicals influencing AQPs is far from complete. Studies are also warranted to confirm in humans the results that have been obtained *in vitro* or with the use of rodent models also taking into account the gender dimorphism that may exist in terms of AQP expression and localization (Rodríguez et al., 2015). It should also be considered that bioactive phytocompounds are often not active *per se*, while their metabolites are. Thus, new and potentially important translational acquisitions are anticipated in the near future, in this exciting field.

## Author contributions

AT and EG contributed to article design, bibliographic search, writing, illustrations, and critical discussion. GT contributed to writing and bibliographic enrichment. CB provided critical discussion; PP contributed to writing and provided critical discussion; RM contributed to article design and writing, and provided critical discussion; GC designed and wrote the first draft of the article and contributed to article refinement, illustrations, bibliographic enrichment, and critical discussion. All authors approved the final version for submission.

### Conflict of interest statement

The authors declare that the research was conducted in the absence of any commercial or financial relationships that could be construed as a potential conflict of interest.
